# Mortality Prediction in Hospitalized COPD Patients Based on FEV_1_/FVC Severity Staging

**DOI:** 10.3390/jcm14217766

**Published:** 2025-11-01

**Authors:** Eduardo Garcia-Pachon, Lucia Zamora-Molina, Carlos Baeza-Martinez, Sandra Ruiz-Alcaraz, Paula Bordallo-Vazquez, Francisco J. Perez-Remacho, Ana Ibarra-Macia, Marta Galan-Negrillo, Justo Grau-Delgado

**Affiliations:** 1 Section of Respiratory Medicine, Hospital General Universitario de Elche, 03203 Elche, Spain; 2 Foundation for the Promotion of Health and Biomedical Research of the Valencian Community (FISABIO), 46020 Valencia, Spain

**Keywords:** chronic obstructive pulmonary disease, GOLD, hospitalization, mortality, prognosis, severity of illness index

## Abstract

**Background**: The recently proposed Staging of Airflow Obstruction by Ratio (STAR) system classifies severity based on the FEV_1_/FVC ratio, potentially offering improved prognostic performance. This study aimed to evaluate the prognostic performance of STAR in patients hospitalized for COPD exacerbation. **Methods**: A retrospective observational single-center study was conducted including COPD patients who were discharged after hospitalization for a severe exacerbation at a university hospital. The clinical and spirometric data in a stable condition, GOLD classification, STAR system, and mortality outcomes were recorded. **Results**: A total of 197 patients (23% female) were included. The follow-up was performed for a minimum of 38 months or until death if it occurred earlier. During the study period, 91 patients died (46%). Patients were distributed according to the STAR classification as follows: 21% in STAR 1, 32% in STAR 2, 28% in STAR 3, and 19% in STAR 4. The agreement between STAR and GOLD was fair (Cohen’s kappa = 0.28), with a moderate correlation (Tau-b = 0.49, *p* < 0.001). STAR grades 2 to 4 demonstrated progressively increasing mortality, while STAR grade 1 showed a mortality similar to grade 2. STAR showed a trend toward a superior discrimination for mortality than GOLD (AUC 0.63 [95%CI 0.55–0.71] vs. 0.55 [0.47–0.63]; *p* = 0.055), although BODEx remained the most accurate predictor (AUC = 0.70 [0.63–0.77]). **Conclusions**: The STAR system effectively stratified the mortality risk among hospitalized COPD patients across grades 2 to 4. However, STAR grade 1 failed to differentiate patients with a lower risk. Although STAR may underestimate severity in individual patients with relatively preserved ratios, its integration into clinical evaluation could enhance prognostic assessments.

## 1. Introduction

Chronic obstructive pulmonary disease (COPD) is a progressive respiratory disorder associated with a risk of exacerbation and mortality, with an accurate assessment of its severity being essential for the optimization of treatment strategies and the appropriate allocation of medical resources. The Global Initiative for Chronic Obstructive Lung Disease (GOLD) classification is based on the percentage of predicted forced expiratory volume in 1 s (FEV_1_) and has been widely adopted for such assessments [1].

The use of the FEV_1_ for grading airflow obstruction is controversial. It is based on equations that have received criticism regarding their applicability in diverse populations and that are derived from pre-bronchodilator data, when they are applied according to post-bronchodilator values [2,3]. Recently, Bhatt et al. [3] proposed an alternative approach that uses the airflow obstruction (FEV_1_/FVC), and not the FEV_1_, to grade severity—the Staging of Airflow Obstruction by Ratio (STAR) system. This system classifies the patients into four grades, STAR 1 with a ratio of 60–70%; STAR 2, with a ratio of 50% to <60%; STAR 3, with a ratio of 40% to <50%; and STAR 4, with a ratio of <40%. Bhatt et al. [3] found that the STAR system better differentiated patients’ symptoms, disease burden, and prognosis than the GOLD scheme, with less sensitivity to demographic characteristics.

The STAR system has also been evaluated for its ability to distinguish mortality in COPD patients. This staging has been demonstrated to have a good performance in distinguishing mortality among stable patients with COPD at different stages [3]. In general, there was a moderate to fair agreement between the STAR and GOLD systems for mortality prediction [4,5]. However, this was only true for grades 2 to 4 of both scales; for grade 1, STAR but not GOLD had a higher mortality than non-obstructed people [3,4,5,6]. The same result was obtained when comparing the ERS/ATS Chronic Obstructive Pulmonary Disease Severity Classification based on z-scores for severity classification instead of GOLD [7].

Patients with COPD requiring hospitalization often present with distinct characteristics (age and comorbidities) that significantly influence their mortality risk [8]. The performance of the STAR system has not previously been evaluated for the risk stratification of mortality in hospitalized patients for COPD. The aim of this study was to assess the performance of STAR in this patient population. We hypothesize that the STAR system will be capable of effectively differentiating distinct mortality risk trajectories in patients with COPD who require hospitalization.

## 2. Materials and Methods

We conducted a retrospective observational analysis of data from patients consecutively admitted to the Pulmonology ward of a university hospital for acute exacerbation of COPD between July 2019 and December 2021. The inclusion date was defined as the date of hospital discharge from the index admission. The follow-up period extended until 28 February 2025.

The diagnosis of COPD was based on age ≥ 40 years, current or past smoking history (≥10 pack-years), and post-bronchodilator FEV_1_/FVC < 70%. Patients with concurrent cancer or interstitial lung disease were not included. Those patients who could not be followed up during the established period were excluded. When patients required more than one admission during the inclusion period, only the data from the first admission were recorded.

Upon admission, the following variables were recorded: age, sex, body mass index (weight [kg]/height^2^ [m]; BMI), smoking history, spirometric values (obtained from the spirometry performed during a stable clinical phase, closest to the date of hospital admission, within 6 months before or after), and number of severe exacerbations (hospitalizations and emergency department visits in the previous five years). Baseline dyspnea (in stable condition) was assessed using the modified Medical Research Council scale (mMRC) [9]. The BODEx index (BMI, airflow obstruction, dyspnea, and severe exacerbation) was calculated for each patient based on standard criteria [10].

GOLD stages 1–4 were based on post-bronchodilator FEV1 of ≥80%, ≥50–80%, ≥30–50%, and <30% [1]. The Staging of Airflow Obstruction by Ratio (STAR) system, with four grades, was established as follows: STAR 1 with a ratio of 60–70%; STAR 2, 50% to <60%; STAR 3, 40% to <50%; and STAR 4, <40% [3].

Date and cause of death were determined using data from the electronic medical records. Survival time was calculated in months.

### 2.1. Ethical Considerations

This study was conducted in accordance with the ethical principles of the Declaration of Helsinki and received approval from the Clinical Research Ethics Committee of the Elche Health Department General Hospital (approval number: PI 31/2019). Written informed consent was obtained from all participants before their inclusion in the study.

### 2.2. Statistical Analysis

Student’s *t* test, analysis of variance (ANOVA), and the Mann–Whitney U test were used when appropriate. Categorical variables were assessed using the chi-squared test. To assess the agreement between measurements, Cohen’s kappa statistic was employed. Correlation analysis was performed using Kendall’s tau-b. Survival analysis was performed using the Kaplan–Meier method, with comparisons using the log-rank test. The discriminatory ability of mortality prediction models was evaluated by assessing the area under the curve (AUC) of the receiver operating characteristic (ROC) curves. The difference between the AUC was compared with the DeLong test. Statistical analysis was performed using the “R” software (version 4.4.2) (https://www.r-project.org/).

## 3. Results

A total of 201 patients were hospitalized due to COPD exacerbation during the inclusion period. Four patients were excluded from the study due to incomplete follow-up (due to relocation to another country in all cases). All four excluded patients were male, with an age range of 62 to 76 years and an FEV1 range from 42 to 59%. The final cohort consisted of 197 patients with COPD who experienced at least one hospitalization for exacerbation. The follow-up ranged from a minimum of 38 months to a maximum of 68 months or until death if it occurred during that time.

Table 1 summarizes the characteristics of the patients. Of the 197 individuals included, 45 were women (23%). Mortality was assessed at one year, three years, and at the end of the study. Among surviving patients, the follow-up duration was 54 months (median, interquartile range [IQR] 43–61), and it was 24 months (median, IQR 11–37) among deceased patients. The number and percentage of survivors and deceased patients at 1 year, 3 years, and at the study end were 166/31 (16% of mortality); 130/67 (34%); and 106/91 (46%).

During the study period, 91 patients (46% of the cohort) died—25% of women and 53% of men (*p* < 0.001). Those who died were older, had poorer pulmonary function, more severe baseline dyspnea, and a higher number of hospitalizations in previous years. These patients also had a higher score on the BODEx multidimensional prognostic index. Patients were distributed as follows according to the STAR classification: 21% in STAR 1, 32% in STAR 2, 28% in STAR 3, and 19% in STAR 4. The distribution of patients according to the GOLD classification was as follows: 4.6% in GOLD 1, 33% in GOLD 2, 47.7% in GOLD 3, and 14.7% in GOLD 4. Survivors and non-survivors differed significantly in their mean STAR score but not in their GOLD classification (Table 1). The agreement between STAR and GOLD showed a Cohen’s kappa statistic of 0.28.

**Figure 1:**
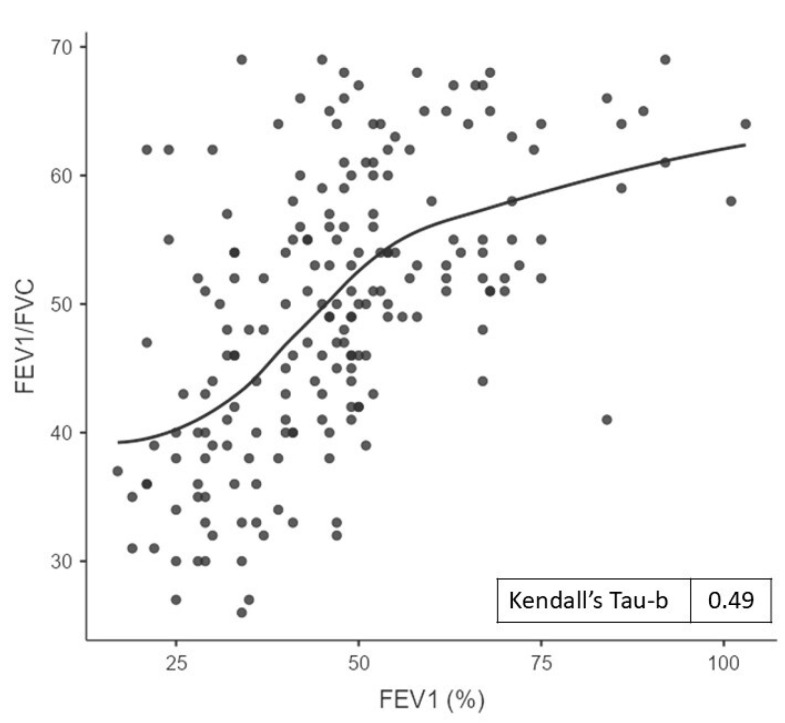
Plot of FEV_1_/FVC against FEV_1_ value.

A significant correlation was observed between the STAR and GOLD COPD severity classification systems (Kendall’s Tau-b value = 0.49, *p* < 0.001). Figure 1 illustrates the correlation between the FEV_1_/FVC and FEV_1_. A significant correlation was observed between the STAR classification and the number of severe exacerbations in the five previous years (Kendall’s Tau-b value = 0.17, *p* < 0.001). This correlation was slightly weaker than that observed between the GOLD classification and the number of prior severe exacerbations (Kendall’s Tau-b value = 0.21, *p* < 0.001).

**Figure 2:**
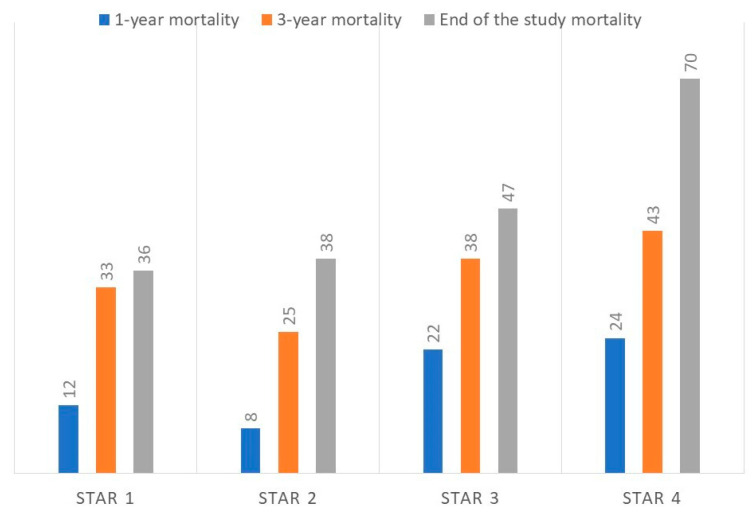
Percentage of deceased patients by STAR category.

Table 2 presents the characteristics of patients stratified by the STAR classification categories. Patients showed greater clinical and functional impairment with increasing STAR classification. The comorbidity [11] did not differ among the various subgroups of STAR patients (Table S1 in the Supplementary Material). The mortality during this study showed different trajectories according to the STAR grading. In groups 2 to 4, mortality increased progressively with both severity and time. The mortality rate of group 1 was similar to that of group 2. Table 2 details the number of surviving and deceased patients for each STAR grade. Figure 2 shows the percentage of deceased patients in each category.

**Figure 3:**
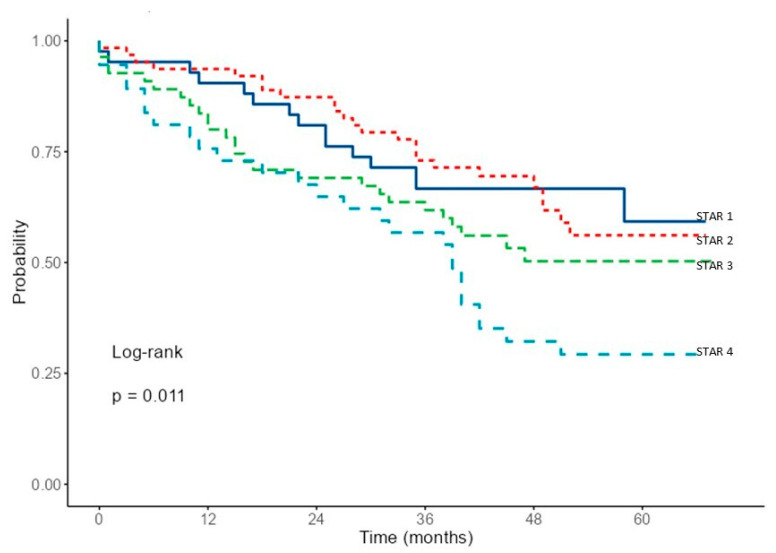
Survival curves for STAR based on Kaplan–Meier estimates.

The survival analysis performed using Kaplan–Meier curve estimates (Figure 3) reveals the distinct mortality trajectories for each STAR grade. While clear differences are observed in grades 2, 3, and 4, grade 1 does not show a trajectory distinct from grade 2. The survival analysis of the groups defined by GOLD showed no significant differences (log-rank = 0.3) (Figure S1 in Supplementary Material).

A significant difference in the cause of death was observed between the STAR groups: 54% of patients in STAR groups 1 and 2 died from a respiratory cause, compared to 82% of patients in STAR groups 3 and 4 (*p* = 0.004). Detailed disaggregated data are presented in Supplementary Material Table S2.

**Figure 4:**
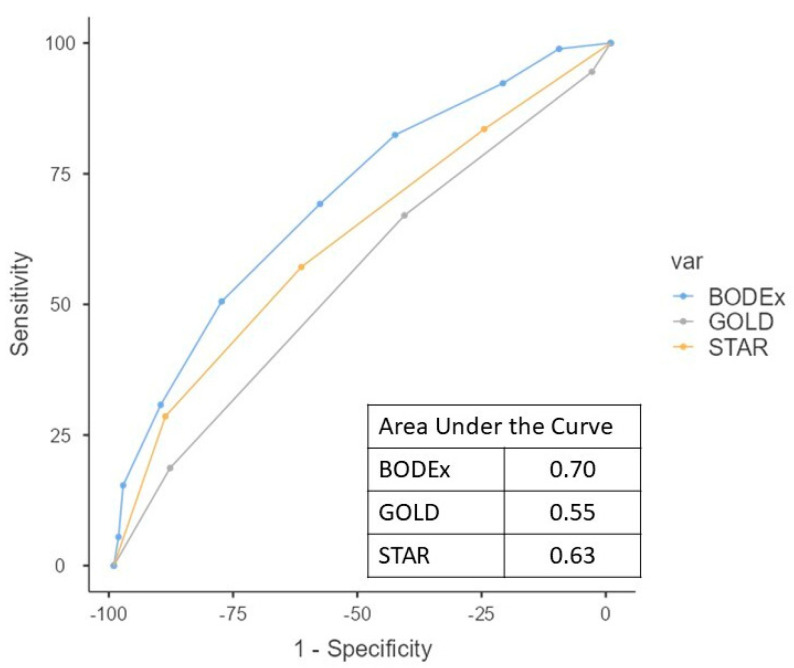
Area under the curve of the receiver operating characteristic (ROC) analysis for mortality classification.

The AUC of the ROC curve for the 1-year survival was 0.62 for STAR and 0.61 for GOLD; for 2-year survival it was 0.61 and 0.57, respectively; and for 3-year survival it was 0.56 and 0.52. The ability of the STAR grade to classify patients according to total mortality tends to be superior to that shown by GOLD (area under the curve 0.63 (95% confidence interval 0.55–0.71) and 0.55 (0.47–0.63), respectively; *p* = 0.055) (Figure 4). The BODEx index showed an AUC of 0.70 (0.63–0.77) (*p* = 0.041, compared to STAR). The power analysis indicated a statistical power of 90% for the observed AUC.

## 4. Discussion

This study demonstrates that the COPD classification system based on the FEV_1_/FVC ratio (STAR grading) is capable of establishing distinct mortality trajectories in patients who have required hospitalization for this disease. This ability has already been described in large series of COPD patients evaluated in other clinical settings [3,4]. However, patients requiring hospitalization for acute exacerbations of COPD constitute a group at a higher risk of mortality and with different causes of mortality compared to less severe cases [12]. Therefore, it was necessary to evaluate whether this ratio allowed for the classification of patients into groups with prognostic capacity.

It is reasonable to hypothesize that in patients with more severe forms of COPD, such as those requiring hospitalization for acute exacerbation, the FEV_1_/FVC ratio may not be as informative as it is in other patient populations. Patients with severe COPD may have a relatively preserved FEV_1_/FVC ratio due to the underestimation of the vital capacity by the forced vital capacity in the presence of an increased collapsibility of the small airways [13]. Furthermore, the premature termination of expiration during spirometric maneuvers could potentially affect the measured values. In our study, however, the tests were conducted by appropriately trained personnel, which minimized the possibility of this technical risk. Air trapping, characterized by an increase in the residual volume (RV) and RV/total lung capacity ratio, is the mechanism that explains the relatively preserved FEV_1_/FVC [14]. Despite proving useful to discriminate a group of subjects at greater risk of a negative outcome, STAR may end up underestimating the severity of the airflow obstruction in individual patients showing worse air trapping and more advanced COPD [15].

Despite this potential limitation, in this study the STAR system demonstrated the ability to differentiate prognostic trajectories. However, an exception was observed in group 1, which, despite theoretically having the mildest obstruction, did not exhibit better outcomes than group 2. This observation might be due to the fact that patients in group 1 are not actually patients with less obstruction but rather patients in whom air trapping or with a concomitant restrictive defect does not allow for their proper classification using the FEV_1_/FVC ratio. In fact, our patients in STAR groups 1 and 2 differed relatively little in FEV_1_; moreover, patients in group 1 had a higher BMI, which may account for a reduction in the FVC.

The STAR system shows only a fair agreement with the GOLD classification. A study found a Cohen’s kappa of 0.25, indicating low concordance [16], and this value was 0.28 in our patients. In this study, a significant correlation was observed between the STAR and GOLD COPD severity classification systems (Tau value 0.49). Previously, Backman et al. [5] had also reported a significant correlation (Tau value 0.45), similar to that obtained in our sample, in a study in the general population.

We conducted an AUC analysis of the ROC curves to compare the mortality prediction capability of STAR and GOLD. In our series, STAR demonstrated a trend toward a slightly greater predictive ability for mortality than GOLD (AUC 0.63 vs. 0.55, the result approached statistical significance with *p* = 0.055). It has been described that, in general, between GOLD and STAR the ability to predict mortality is similar in non-hospitalized patients [4,5]. In our series with patients demonstrating greater clinical severity, the trend seems to indicate that STAR might have a better predictive ability. In previous studies that assessed the predictive value of different versions of the GOLD 1–4 system for all-cause mortality, the AUC ranged between 0.56 and 0.63 [17,18].

The STAR grading system has demonstrated less promising results for predicting exacerbations in COPD patients. It seems that STAR does not perform as well as the GOLD classification in predicting exacerbation risk. Specifically, the STAR system did not show significant differences in the risk of exacerbations between its different stages [3,19]. Our findings, from a retrospective analysis of severe exacerbations, are consistent with these prior data. While both the STAR and GOLD grades correlated with the number of COPD exacerbation-related hospitalizations in the preceding five years, this correlation was modestly stronger with the GOLD classification.

In this study, we included the BODEx index (a multidimensional instrument specifically developed for prognostic assessment), which demonstrates a superior predictive capacity for mortality. Although STAR and GOLD have been assessed as mortality predictors, their predictive accuracy, as expected, is inferior to that of this instrument. Indeed, it had already been observed that the BODE index showed superior predictive capability when compared with GOLD [18]. The BODEx index is one of the proposed variations in the BODE index that has demonstrated a great predictive ability [10]. STAR and GOLD are prognostic classification systems based on basic functional data, while BODEx is a multicomponent index specifically designed to predict mortality. The assessment of their different results should be interpreted taking these characteristics into account. There are various other multidimensional indices that manage to predict the prognosis of patients with COPD [20,21] and whose objective is different from the classifications intended with the GOLD and STAR classifications.

As an additional finding in our study, we observed that the cause of mortality also differs according to the patients’ STAR category. Patients with less severe obstruction (STAR 1 and 2) showed a higher proportion of deaths from non-respiratory causes compared with those with greater obstruction (STAR 3 and 4).

This study presents certain limitations. Conducted at a single center, is a retrospective study that includes a relatively small number of patients, which precludes multivariate analysis. A significant limitation of our study is the inability to perform a multivariate analysis. While our unadjusted results indicate that STAR defines distinct prognostic trajectories, we cannot confirm that it is an independent predictor of mortality due to the potential influence of uncontrolled confounding variables. It exclusively included COPD patients with a history of tobacco smoking, thereby precluding the attribution of its potential utility to other forms of COPD. Furthermore, pulmonary volume studies were not available to ascertain the impact of air trapping on the analyzed outcomes. This information would have been particularly insightful in understanding the unexpected behavior observed in group 1. Nevertheless, this sample has yielded useful information for the stated objectives. In addition, although the short-term repeatability of spirometry in stable COPD is generally high—with intraclass correlation coefficients for FEV_1_ and FVC often exceeding 0.9—absolute session-to-session differences can nonetheless be considerable [22], potentially influencing both the acquisition and interpretation of results within the STAR classification. Probably, incorporating the STAR grading system into COPD evaluation may enhance the precision of disease severity assessments and improve patient management by providing a more detailed understanding of the disease progression and outcomes. Nevertheless, these findings are preliminary, and larger-scale studies are warranted to confirm these findings and further validate the prognostic utility of the STAR system.

## 5. Conclusions

In conclusion, this study observed that in patients requiring hospitalization for COPD, the STAR system, based on the degree of obstruction (FEV_1_/FVC), is capable of establishing distinct prognostic trajectories for mortality in grades 2 through 4. However, its ability to differentiate patients in group 1 has not been established. When compared to the GOLD classification, STAR demonstrated a clinically meaningful trend toward the improved prediction of mortality (while not statistically significant), particularly in moderate-to-severe cases. This positions STAR as a promising tool for risk assessment in hospitalized cohorts, where accurate prognostication is critical.

## Figures and Tables

**Table 1:** Patients’ characteristics.

	Total	Survivors	Deceased	*p* Value
Number of patients	197	106	91	
Female, n (%)	45	34 (75%)	11 (25%)	
Male, n (%)	152	72 (47%)	80 (53%)	
Age (years)	73 ± 10	70 ± 9	77 ± 9	<0.001
Current smokers, n (%)	80 (41%)	49 (61%)	31 (39%)	0.08
Pack-years	58 ± 26	53 ± 23	63 ± 28	0.006
FVC % predicted	73 ± 20	74 ± 19	72 ± 20	0.34
FEV_1_% predicted	47 ± 17	49 ± 16	45 ± 18	0.08
FEV_1_/FVC, %	50 ± 11	52 ± 10	48 ± 11	0.007
Dyspnea (mMRC scale)	2.2 ± 0.9	1.9 ± 0.9	2.5 ± 0.8	<0.001
Severe exacerbations in the past 5 years, n	1.03 ± 1.54	0.65 ± 1.26	1.46 ± 1.73	<0.001
BODEx	3.7 ± 2.0	3.0 ± 1.8	4.5 ± 1.9	<0.001
GOLD	2.7 ± 0.8	2.7 ± 0.7	2.8 ± 0.8	0.20
STAR	2.4 ± 1.0	2.2 ± 0.9	2.7 ± 1.1	0.001

Values are mean ± SD, unless otherwise specified.

**Table 2:** Patient characteristics and mortality according to STAR grades.

STAR Grade	1	2	3	4	*p* Value
Number and percentage	42 (21%)	63 (32%)	55 (28%)	37 (19%)	
Age, years	70 ± 11	74 ± 10	73 ± 11	74 ± 8	0.29
Severe exacerbations, n	0.67 ± 1.07	0.76 ± 1.30	1.13 ± 1.63	1.73 ± 2.04	0.025
Dyspnea, mMRC	1.8 ± 0.9	2.0 ± 0.9	2.2 ± 0.9	2.8 ± 0.6	<0.001
FEV_1_, % of reference	58 ± 18	53 ± 15	44 ± 11	31 ± 8	<0.001
FEV_1_/FVC, %	64 ± 3	54 ± 3	45 ± 3	34 ± 4	<0.001
Body mass index	31 ± 7	28 ± 6	26 ± 5	24 ± 4	<0.001
1-year mortality	37/5 (12%)	58/5 (8%)	43/12 (22%)	28/9 (24%)	0.07
3-year mortality	28/14 (33%)	47/16 (25%)	34/21 (38%)	21/16 (43%)	0.27
Mortality at the end of the study	27/15 (36%)	39/24 (38%)	29/26 (47%)	11/26 (70%)	0.007

Values are mean ± SD. Values of mortality represent number of survivors/deceased, with corresponding mortality percentage in parentheses.

## Data Availability

Data are available from the corresponding author upon justified request.

## Supplementary Materials

The following supporting information can be downloaded at https://www.mdpi.com/article/10.3390/jcm14217766/s1. Figure S1. Survival analysis of GOLD classification. Table S1. Comorbidities according to STAR categories. Table S2. Causes of mortality according to STAR categories.

## Abbreviations

The following abbreviations are used in this manuscript:

COPD	Chronic Obstructive Pulmonary Disease
GOLD	The Global Initiative for Chronic Obstructive Lung Disease
FEV_1_	Forced Expiratory Volume in One Second
FVC	Forced Vital Capacity
STAR	Staging of Airflow Obstruction by Ratio
