# Supplementary material for: Mortality Prediction in Hospitalized COPD Patients Based on FEV1/FVC Severity Staging
Source: J Clin Med. 2025 Nov 1;14(21):7766. doi: 10.3390/jcm14217766 (PMC12610859; doi:10.3390/jcm14217766)
Supplement: Supplementary file 1 [file jcm-14-07766-s001.zip › jcm-3951695-supplementary.pdf]

Supplementary Material

Table S1. Comorbidities according to STAR categories

One-Way ANOVA (Welch's)

	F	df1	df2	p
**Charlson**	1.80	3	53.2	0.159

Group Descriptives

	STAR	N	Mean	SD	SE
**Charlson**	**1**	18	2.67	1.28	0.302
	**2**	37	2.03	1.09	0.180
	**3**	30	1.83	1.02	0.186
	**4**	31	2.06	1.09	0.196

Ondeck NT, Bohl DD, Bovonratwet P, et al. Discriminative ability of commonly used indices to predict adverse outcomes after poster lumbar fusion: a comparison of demographics, ASA, the modified Charlson Comorbidity Index, and the modified Frailty Index. *Spine J*. 2018; 18: 44-52.

Figure S1. Survival analysis of GOLD classification.

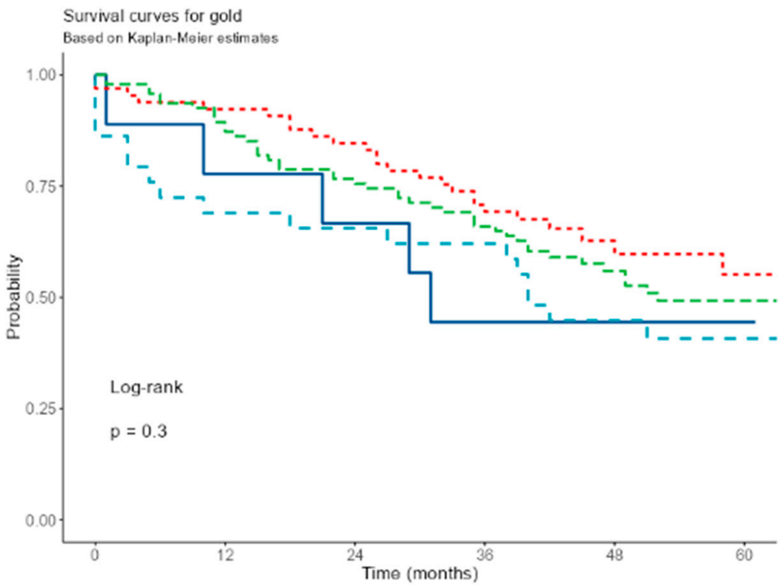

Table S2. Causes of mortality according to STAR categories

	Respiratory causes	Non-respiratory causes
STAR 1	COPD, 5<br>Lung cancer, 1	Other cancers, 2<br>Heart failure, 3<br>Renal failure, 2<br>Infection, 1
STAR 2	COPD, 8<br>Lung cancer, 4<br>Pneumonia, 2	Unknown, 1<br>Other cancers, 1<br>Heart failure, 4<br>Infection, 3<br>Stroke, 1
STAR 3	COPD, 16<br>Lung cancer, 1<br>Pneumonia, 3	Unknown, 1<br>Other cancers, 1<br>Coronary heart disease, 2<br>Renal failure, 1<br>Intestinal ischemia, 1
STAR 4	COPD, 16<br>Lung cancer, 4<br>Pneumonia, 2	Heart failure, 2<br>Coronary heart disease, 1<br>Stroke, 1
